# Confusion Arising from too many Messages in Health Awareness Campaigns in Rural Communities in Malawi

**DOI:** 10.1007/s10900-026-01617-3

**Published:** 2026-07-25

**Authors:** Chiara Pittalis, Francesca Munthali, Josephine Muhasuwa, Mary Tembo, Zsofia Torok, Antonio Jaén Osuna, Chrissy Banda, Erik Schouten, Jakub Gajewski

**Affiliations:** 1 Institute of Global Surgery, School of Population Health, RCSI University of Medicine and Health Sciences, Dublin, Ireland; 2 Agency for Scientific Research and Training, Lilongwe, Malawi; 3 Blantyre District Health Office, Ministry of Health, Blantyre, Malawi; 4 Centre for Global Surgery, Stellenbosch University, Cape Twon, South Africa

**Keywords:** Health promotion, Breast cancer, Reduce premature mortality, Multi-stakeholder cooperation, Developing countries

## Abstract

Health talks and community engagement are central to many public health programmes, yet their effectiveness is shaped by complex social, cultural, and informational contexts. Even well-designed interventions can be undermined when communities receive overlapping health messages, leading to confusion, competing interpretations, or message fatigue. Drawing on experiences from a breast health and breast cancer awareness campaign delivered through the Akazi Project in rural Malawi, this commentary reflects on lessons from community engagement and their implications for health communication. Between October and December 2025, breast health education was delivered across eight rural communities through participatory sessions involving more than 300 community leaders, including chiefs, religious leaders, youth volunteers, and women’s groups. Discussions consistently revealed a strong demand for basic information on breast health, clarification of common misconceptions, and guidance on how breast cancer messages aligned with advice from HIV, cervical cancer, and vaccination programmes. These experiences highlighted the need for greater coordination across health programmes and for communication approaches that acknowledge how people interpret new information alongside existing public health messages. We argue that integrated messaging, stronger collaboration between programmes, and opportunities for community feedback should be central components of health communication strategies, particularly in rural settings where limited health literacy intersects with exposure to multiple health initiatives.

## Introduction

Community engagement through health talks has long been a cornerstone of social behavioural change communication (SBCC) for health improvement across much of sub-Saharan Africa [1] including in breast cancer care [2]. They are widely recognised for their many benefits: creating awareness, addressing myths, and bridging the gap between communities and formal health systems [3, 4]. Yet achieving real and sustained change through these strategies remains a complex challenge [3, 5]. Success depends on a deep understanding of the audience, as well as on substantial investment in making messages culturally relevant, locally appropriate, and aligned with known behaviour-change triggers [1, 6]. Still, the outcomes of health communication are never entirely predictable [4], as people are shaped by diverse experiences, beliefs and social contexts that influence how messages are received and acted upon [7]. In addition, the effects of overexposure to health campaigns, including message fatigue and other associated risks, are unknown as there is paucity of research in this area [8, 9].

Our recent fieldwork delivering a breast health and breast cancer awareness campaign in rural Malawi brought these issues into sharp focus. This experience prompted reflection not only on persistent gaps in essential health knowledge, but also on a broader systemic issue: the cumulative effects of multiple, siloed health messages circulating within the same communities. In such crowded communication environments, even well-designed messages risk becoming blurred or misunderstood. In this commentary, we reflect on these experiences and the lessons they offer for health promotion practice in breast cancer and beyond.

## Health Information Sharing

Our campaign was undertaken as part of the Akazi Project, a multi-stakeholder partnership involving academics, sectoral experts, local health authorities and clinicians aimed at strengthening breast cancer care in Malawi and reducing premature mortality. Breast cancer survival rates are extremely low in the country [10], largely due to late‑stage presentation driven by limited health literacy, sociocultural norms, stigma, and poor awareness of available care services [11–13]. These social and behavioural barriers are particularly pronounced among rural dwellers, who comprise the majority of the population. Hence, the campaign aimed to improve knowledge of breast conditions and cancer symptoms, influence care‑seeking norms, and increase uptake of diagnostic services in view of encouraging earlier identification of symptomatic cases and improving chances of successful treatment.

It was grounded in extensive preparatory work and co‑designed with community engagement specialists and the District Health Office (DHO), ensuring messages were culturally resonant, contextually appropriate, and aligned with local realities. It was delivered between October and December 2025 across eight rural communities in Southern Malawi. Implemented through local health centres and their catchment areas, the campaign enabled coordinated engagement at both facility and community levels. Over 300 community leaders - including religious leaders, village chiefs, youth volunteers, Health Advisory Committee members, and women’s groups - were oriented and mobilised as trusted messengers to promote breast health awareness and timely health‑seeking within their respective communities. Orientation sessions were highly participatory, incorporating interactive activities, such as music (through joint singing), storytelling and practical demonstrations, and deliberately created space for questions and open discussion.

Community questions were systematically documented and thematically analysed to identify and address priority concerns, and guide subsequent Akazi Project activities. A visual summary is provided in Table 1 below.

**Table 1:** Key questions raised by community members

Core area of concern for community members	Broad topics	Specific topics	Specific details
Nature of breast cancer and link with or distinction from other cancers			
Causes, risks and transmission	• Role of genetics		
	• Lifestyle related risks	o Smoking, alcohol consumption and body weight	
	• Reproductive behaviours and their link with breast cancer	o Sexual activity	
		o Family planning	- Risk associated with early menstruation & giving birth at young age - Risk associated with giving birth at older age
		o Breastfeeding	- Infections and breast cancer - Risk of cancer from baby burping or regurgitating on mother’s breast - Transmission to baby through breastfeeding
	• Other transmission mechanisms	o Sharing blades	
		o Using second hand bras	
		o Bacterial or viral causes	- Existence of vaccines for prevention
	• Implications for other family members		
Signs and symptoms recognition	• How to recognise signs of breast cancer	o When early, not obvious signs	
		o When breastfeeding (e.g. pain and infections common)	
	• Self-breast examination	o Who should do it, when & role of husband	
Understanding available services	• What to do when signs of breast cancer are noticed		
	• Diagnostic process, timing and cost		
	• General services and supports available	o Role of Queen Elizabeth Central Hospital	
		o Financial support	
Further raising community awareness			

The questions raised were largely foundational, ranging from breast cancer aetiology, signs and symptoms, to availability of services locally and at the nearest specialist hospitals, Queen Elizabeth Central Hospital (QECH). They also showed practical concerns, e.g. implications of breast cancer for breastfeeding or cost of care for disadvantaged families.

While the questions reflected the novelty of breast cancer as a concept within these communities, they also highlighted the presence of widespread beliefs, such as that breast problems may be caused by a baby regurgitating milk onto the mother’s chest during breastfeeding, by wearing second-hand bras purchased at the market or by witchcraft.

These misconceptions, not unique to Malawi, are a reflection of sociocultural factors and limited access to structured health information [14–16]. Life in most rural areas is shaped by the rhythm of the seasons, the cycle of planting and harvest, and the gatherings of markets, family events, and religious celebrations. In these settings, information travels through rich and deep social channels: the radio, conversations at the borehole or the market, community meetings, and local churches, often enriched by oral traditions passed down through generations, and news from people returning from towns or other regions [17].

As such, health information becomes part of a dynamic tapestry of local narratives, beliefs and experiences shared through these social networks [6]. This can be a powerful process to circulate health information, but it can also make it difficult to distinguish which messages are true, partly so or mistaken [18] - especially when information on similar topics from different sources appears equally credible or is delivered with similar authority.

## The Effect of Multiple, Uncoordinated and Insufficiently Prioritised Health Messages

Another layer to this complexity is added by the proliferation of international and local health programmes operating in parallel across rural Malawi, all relying heavily on health talks and community engagement as a part of their SBCC efforts across multiple health areas - from HIV [19–21] and malaria [22], to non-communicable diseases, including cervical cancer [23, 24], and vaccination campaigns, e.g. Human Papillomavirus (HPV) [25], just to name a few.

While these initiatives are helpful, they often deliver overlapping or fragmented messages, with the potential of creating confusion among the target communities and diluting impact.

Examples of this in practice were evident during our sessions, as many questions raised by community members appeared influenced by exposure to previous health talks on HIV and cervical cancer. For instance, several participants across different communities asked whether breast cancer was caused by a virus or bacteria, whether it could be passed on through breastfeeding, sexual activity or sharing razor blades, and whether a vaccine existed to prevent it.

Many of these questions were simply information-seeking, but others reflected genuine confusion - as in this instance:

*We have been informed about womb cancer*,* cervical cancer*,* prostate cancer*,* and now breast cancer - all seem related to intimacy. Does this mean that sexual activity between husband and wife is harmful?*

Another issue seemed to be the content of the health messages. For example, one participant made this point:

*For cervical cancer*,* we are told that giving birth too young increases the risk*,* yet now you are saying that giving birth late increases the risk of breast cancer. These messages seem contradictory. What*,* then*,* is the right age to give birth?*

Although the information regarding both cervical and breast cancer is accurate, this example suggests that presenting risk factors without clear and coordinated behavioural guidance may lead to frustration rather than providing respondents with an actionable health message.

Despite these observations, the Akazi sessions were generally well received, and community leaders expressed interest in exploring ways to disseminate this knowledge more widely within their communities and to collaborate more closely with health providers. The challenge remains how to achieve this while minimising friction with other health programmes and avoiding message fatigue or frustration among community members.

## Why this Matters

Indeed, the field experiences reported in this manuscript point to wider issues well-recognised among the scientific community and development practitioners.

One such issue relates to weaknesses in strategy design in terms of delivery and content. Disease-specific vertical campaigns, which rarely coordinate messaging at the district or community level, have long been criticised for their tendency to operate in silos and limited effectiveness [26, 27].

A further issue is governance and coordination. Many programmes are funding‑ or project‑driven, with no central focal point to coordinate activities across sectors or align them with national priorities. In Malawi, high levels of donor fragmentation and multiple implementing partners have been shown to lead to poor coordination and misalignment with the Ministry of Health planning [27], undermining unified messaging and service delivery. National policy documents similarly note weaknesses in planning and coordination between the Ministry of Health, district health offices, and external partners, with some partners bypassing district oversight altogether [28].

While the potential for improved coordination is more apparent in large, well-established health programmes [26, 29], breast cancer remains a relatively neglected topic. Even the National Cancer Strategy makes only limited reference to it [13], underscoring the need for greater attention. The Strategy does, however, point to opportunities for integration with cervical cancer control efforts, which are already far reaching. Although there are differences - given that breast cancer control in Malawi relies primarily on early identification of symptomatic women rather than population-level screening - the communication and health promotion components of cervical and breast cancer programmes could be delivered jointly. This approach should help leverage existing community engagement channels and reduce duplication of effort.

At the level of programme delivery, many health campaigns rely on community health workers (CHWs) to reach rural populations. CHWs can become overburdened by the multiple demands of parallel health programmes, contributing to high workloads and task multiplicity; in some cases, this may lead them to prioritise activities that are more feasible or more rewarding, rather than supporting all initiatives equitably [30, 31]. In this context, the delivery of health communication risks becoming increasingly transactional, shaped by programme incentives for CHWs.

Collectively, these inefficiencies have tangible consequences for target communities. Poorly designed campaigns, overlapping messages, and overburdened or under-motivated health workers can compromise the quality and consistency of health communication. It is therefore unsurprising that community members experience uncertainty around health messages received and appropriate course of action, as observed in this manuscript.

## What can be Done Differently?

Drawing on our field experience and long-standing engagement in rural areas, we suggest a set of practical mechanisms that may help improve coordination across health programmes and reduce fragmentation in community-level health communication. These are essential for strengthening care and advancing health promotion in the face of the growing breast cancer burden across Sub-Saharan Africa [32], while also supporting broader health promotion efforts in rural communities.

Firstly, early engagement of DHOs (or similar health management authorities) during programme design is critical to align new initiatives with existing activities at district and community levels. Such engagement can improve the visibility of planned interventions and reduce duplication across programmes operating in the same geographical areas [26]. The Akazi Project illustrates this approach to engagement with the DHO, including involvement of cervical cancer officers and specialists at Queen Elizabeth Central Hospital (QECH) to explore opportunities for alignment around shared health promotion priorities. In our experience, this way of working strengthened links between health providers and communities, enhanced the credibility and quality assurance of the intervention, and facilitated its integration into the overall DHO “Masterplan” of community health interventions. This approach is considered good practice for community-level programmes, but is not always adopted by organisations working outside the public health system.

Second, the development of shared communication frameworks could enable non-governmental organisations and implementing partners to deliver more consistent health messages across programmes. Agreed core messages, terminology, and framing - adapted locally but aligned across health areas - may help minimise confusion and improve message coherence for communities exposed to multiple campaigns. In the context of the Akazi Project, we are working to align messages among the DHO, service providers, and NGOs involved in breast cancer awareness. Although still at an early stage, aligning messaging is feasible and necessary.

Third, strengthened training for health educators and community health workers could support more integrated SBCC. Rather than delivering disease-specific messages in isolation, educators could be equipped to link related information across health areas - for example, situating breast health messages within existing cervical cancer or sexual and reproductive health discussions - while remaining sensitive to programme objectives and workload constraints. Such capacity building may require longer-term investment, but it represents an important step towards more coherent health communication.

Finally, more meaningful forms of community engagement are needed [33], moving beyond predominantly top-down information delivery [34]. Participatory approaches that actively involve community members, leaders, and local structures in shaping messages and priorities improve relevance, trust, and uptake, while also providing feedback on how messages are received and understood [35]. In the Akazi Project, we found that creating space for community members to raise questions, collect feedback and integrate it into project activities, was highly valuable. We hope that our positive experience can encourage similar practices in other initiatives.

## Conclusion

Health talks and community engagement remain among the most widely used tools for health promotion in rural settings. Yet, when delivered in isolation, they risk fragmenting rather than reinforcing public understanding. Our experience highlights the importance of harmonising health communication - across diseases where feasible, and across programmes and partners - to build coherent and lasting health literacy.

As health communicators we also have a responsibility to understand the information needs of communities and the most appropriate delivery mechanisms, taking into account the complexity of rural social life, in order to design messages that are relevant, clear, and actionable. This requires moving away from framing communities as passive or uninformed. Such recognition is essential for developing health communication that resonates with, rather than disrupts, the everyday rhythms of community life.

By sharing these experiences, we aim to encourage others to take up this challenge and contribute to the ongoing discussion on how best to strengthen health communication for the benefit of rural communities.

## Data Availability

Not applicable, as this is a commentary. All information referred to in this commentary is presented within the body of the article and its references.
